# Metagenomic Characterisation of Antibiotic Resistance in Anaerobic Digesters and Their Implications for Environmental Health

**DOI:** 10.1111/1758-2229.70400

**Published:** 2026-10-01

**Authors:** Olasunbo Aina Olasupo, Oluwaseyi Paul Olaniyan, Sunday Babatunde Akinde, Ademola Ayodele Adesoye, Fatimah Adenike Ajadi, Omokaro Obire, Elijah Kolawole Oladipo, Waidi Folorunso Sule, Olamilekan Emmanuel Oyinloye, Adedolapo Aishat Salami, Emmanuel Sunday Fajoyegbe, Adejoke Janet Ajani, Olabisi Ojo

**Affiliations:** ^1^ Department of Microbiology Osun State University Osogbo Nigeria; ^2^ Microbial Biotechnology and One Health Research Laboratory, Osun State University Osogbo Nigeria; ^3^ Multidisciplinary Research Laboratory Osun State University Osogbo Nigeria; ^4^ Department of Microbiology Federal University of Health Sciences Ila‐Orangun Nigeria; ^5^ Department of Microbiology Rivers State University Port Harcourt Nigeria; ^6^ Molecular Biology, Immunology and Bioinformatics Laboratory, Department of Microbiology Adeleke University Ede Nigeria; ^7^ Genomics Unit Helix Biogen Institute Ogbomoso Nigeria; ^8^ Department of Natural Sciences Albany State University Albany Georgia USA

**Keywords:** anaerobic digestion, environmental AMR surveillance, environmental resistome, microbial functional profiling, waste‐to‐energy systems

## Abstract

Antimicrobial resistance (AMR) in environmental systems represents an emerging One Health challenge, with anaerobic digesters potentially serving as reservoirs of resistance‐associated genetic features. This study analysed four publicly available anaerobic digester shotgun metagenomic datasets (M2, M3, M12 and M17) retrieved from the NCBI Sequence Read Archive to characterise microbial functions, resistance‐associated annotations and antibiotic target loci. The datasets were generated using Illumina HiSeq 2000 sequencing and analysed through quality assessment, assembly, functional annotation and resistome profiling. A total of 1082 resistance‐associated annotations were identified, with the highest abundance detected in M2 (348), followed by M12 (276), M17 (259) and M3 (205). Detected annotations were associated with diverse antimicrobial categories, including aminoglycosides, quinolones, beta‐lactams, tetracyclines, glycopeptides, macrolides and sulfonamides. Several frequently detected loci, including *rpoB*, *rpoC*, *gyrA*, *gyrB*, EF‐Tu, EF‐G, Ddl and KasA, were interpreted as antibiotic target or housekeeping loci rather than confirmed acquired resistance genes. Exploratory co‐abundance analysis identified strong associations among functionally linked loci, including *rpoB‐rpoC* and *gyrA‐gyrB*, likely reflecting shared genomic occurrence or functional relationships. These findings highlight the resistome potential of anaerobic digesters while emphasising the need for host‐resolved metagenomics, mobile genetic element analysis and mutation‐level characterisation to clarify environmental AMR risks.

## Graphical abstract

Anaerobic digester metagenomes revealed diverse resistance‐associated annotations and antibiotic target loci, highlighting their potential role in environmental AMR surveillance. The findings emphasise the need for cautious interpretation of resistome profiles and further host‐resolved analyses to determine the mobility and clinical relevance of detected resistance‐associated features.

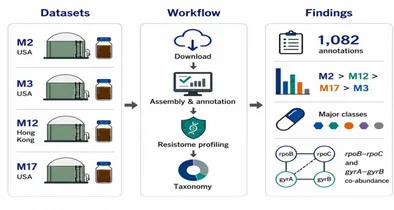

## 1 Introduction

Antibiotic resistance is increasingly recognised as one of the most pressing global health challenges, with far‐reaching consequences for human health, environmental integrity, and animal welfare (Ifedinezi et al. 2024; Muteeb et al. 2023). The proliferation of resistance to commonly used antibiotics contributes to treatment failure, prolonged hospitalisation, and escalating healthcare costs, placing immense strain on health systems worldwide. While the clinical dimensions of antimicrobial resistance (AMR) are well established, its environmental drivers, particularly within agricultural ecosystems, are gaining critical attention.

Agricultural systems have emerged as key reservoirs and transmission pathways for AMR, with sources including effluents from wastewater treatment plants (WWTPs), livestock manure, and, more recently, anaerobic digesters (La Rosa et al. 2025; Uluseker et al. 2021). Anaerobic digestion (AD), widely adopted for treating organic waste such as agricultural residues, food waste, animal waste, and sewage sludge, offers dual benefits of waste management and renewable energy production (Olaniyan et al. 2025; Alengebawy et al. 2024; Olaniyan and Ajayi 2021). However, AD systems may also serve as hotspots for antibiotic resistance genes (ARGs) and mobile genetic elements (MGEs), particularly when digestate is repurposed as fertiliser, potentially facilitating the environmental dissemination of resistance determinants (Allegrini and Zabaloy 2024; Wolak et al. 2023; Lima et al. 2020).

Microbial communities within anaerobic digesters are adept at degrading complex organic substrates; however, they may also harbour diverse resistomes that pose risks to One Health (Lin et al. 2022). Metagenomic sequencing has revolutionised the study of microbial ecology, enabling high‐resolution, culture‐independent profiling of ARGs, MGEs, and functional gene networks (Olsen and Riber 2025; Nogueira and Botelho 2021). In the context of AD systems, metagenomics provides critical insights into microbial interactions and the mechanisms of horizontal gene transfer that underpin the spread of resistance across bacterial populations (Von Wintersdorff et al. 2016). Despite its promise, comprehensive characterisation of the resistome and mobilome in anaerobic digesters remains limited.

Given the expanding global reliance on AD as a sustainable waste treatment strategy, a deeper understanding of the microbial diversity, resistome, and virulome within these systems is urgently needed. This study employs metagenomic sequencing data from the NCBI to investigate the abundance and diversity of ARGs and MGEs in anaerobic digestate samples collected from geographically distinct sites. Our central research question is ‘What is the diversity and abundance of antibiotic resistance genes and mobile genetic elements in anaerobic digester systems, and what are the implications for environmental health and one health?’

Despite increasing concern about antimicrobial resistance in waste‐treatment environments, the contribution of anaerobic digesters to the environmental resistome remains insufficiently resolved. This gap is important because digestate from anaerobic digestion systems is often reused as organic fertiliser, creating a possible route through which resistance‐associated genetic material may enter agricultural soils and wider ecological networks. A clearer distinction is also needed between acquired antibiotic resistance genes and antibiotic target or housekeeping loci, since the detection of target genes alone does not confirm resistance unless resistance‐conferring mutations or mobile gene associations are demonstrated.

By elucidating the microbial dynamics and resistance profiles of anaerobic digesters, therefore, this study aimed to characterise the functional profile, resistance‐associated annotations and co‐abundance patterns of publicly available anaerobic digester shotgun metagenomes, while distinguishing acquired resistance determinants from antibiotic target or housekeeping loci in order to provide a cautious assessment of their environmental and One Health relevance.

## 2 Materials and Methods

### 2.1 Dataset Retrieval From NCBI

The four datasets, with BioSample accession numbers SAMN27412227, SAMN27412222, SAMN03742276 and SAMN27412223, were publicly available whole‐genome shotgun metagenomic sequences deposited in the NCBI Sequence Read Archive (SRA) (SRA; https://www.ncbi.nlm.nih.gov/sra). Sequencing was performed using the Illumina HiSeq 2000 platform, producing short‐read shotgun metagenomic data from anaerobic digester microbial communities. No additional sequencing was performed in the present study. Raw sequence datasets were downloaded and subjected to quality assessment, assembly, annotation, and downstream analyses of the resistome.

The sample codes M2, M3, M12 and M17 were used as internal analytical identifiers for the four selected metagenomic datasets. The non‐sequential numbering reflects the selection of eligible datasets from a wider pool of available SRA records and does not represent a chronological sampling order. The corresponding BioProject, BioSample and SRA accession numbers are provided in Table 1 to ensure traceability and reproducibility.

**TABLE 1:** General characteristics of metagenomic samples from anaerobic digesters.

Bioproject	Biosample	SRA	Sample ID	Size (GB)	Location	Layout	Total reads (GB)	Submitter	Year	Seq. platform
PRJNA824684	SAMN27412227	SRR18681321	M2	1.7	USA: Ithaca	Single	4.7	Univ. of Tuebingen	2022	Illumina HiSeq 2000
PRJNA824684	SAMN27412222	SRR18683126	M3	1.4	USA: Ithaca	Single	3.7	Univ. of Tuebingen	2022	Illumina HiSeq 2000
PRJNA284867	SAMN03742276	SRR2043635	M12	2.2	Hong Kong	Paired	3.4	HKU	2015	Illumina HiSeq 2000
PRJNA824684	SAMN27412223	SRR18683125	M17	1.7	USA: Ithaca	Single	4.7	Univ. of Tuebingen	2022	Illumina HiSeq 2000

Abbreviations: GB = gigabases of sequence data; Layout = library read layout (single‐end or paired‐end); M = Sample ID/Metagenome number; Seq. Platform = sequencing platform; SRA = Sequence Read Archive.

### 2.2 Read Processing and Genome Assembly

Raw sequencing reads were quality‐checked and processed using the BV‐BRC genome assembly service on the Illumina platform to ensure standardised workflows (Gurevich et al. 2013). Assemblies were refined with Unicycler v0.4.8 (Wick et al. 2017), which integrates short‐ and long‐read data, followed by Samtools v1.17 for alignment handling and Pilon v1.2.3 for error correction and gap filling (Antipov et al. 2020). Assembly quality was evaluated with QUAST v5.2.0, which reports genome length, contig number, N50, GC content, and L50. High‐quality assemblies were subsequently used to annotate ARGs and mobile genetic elements (MGEs).

### 2.3 Functional Annotation and Resistome Profiling

#### 2.3.1 Gene Prediction and Functional Categorisation

Functional annotation of assembled contigs was performed using the Rapid Annotations using Subsystems Technology (RASTtk) toolkit within the BV‐BRC pipeline, with bacteria specified as the target domain (Wattam et al. 2024). Annotated coding sequences (CDSs) were subsequently mapped to the Kyoto Encyclopedia of Genes and Genomes (KEGG) database to assign metabolic functions (Xu et al. 2022).

#### 2.3.2 Antibiotic Resistance Gene Identification

Resistance‐associated annotations were identified using the Comprehensive Antibiotic Resistance Database, CARD, implemented through the BV‐BRC annotation pipeline. The exported annotation files were curated to retain entries with documented links to antimicrobial resistance mechanisms, antibiotic target pathways, resistance‐associated gene products or antimicrobial class information. To avoid overinterpretation, retained annotations were further classified into two interpretive groups. The first group comprised putative acquired ARGs, including genes encoding resistance enzymes, efflux systems, target protection proteins or other recognised transferable resistance determinants. The second group comprised antibiotic target or housekeeping loci, including *rpoB*, *rpoC*, *gyrA*, *gyrB*, *EF‐Tu*, *EF‐G*, *Ddl*, *KasA*, *S12p* and related loci, whose presence alone does not confirm resistance without mutation‐level or allele‐level evidence. Duplicate annotations were checked by sample, gene name and functional product before comparative summary. Counts reported in this study therefore represent resistance‐associated annotations detected in assembled metagenomic contigs and should not be interpreted as phenotypic resistance measurements (Abramova et al. 2024).

### 2.4 Taxonomic Profiling

#### 2.4.1 Merging and Pre‐processing of Reads

Assembled reads were concatenated using the Merge. files function on the Galaxy platform (v1.3.1) to generate a single merged FASTA file for downstream taxonomic analysis. The merged reads were then processed to enable accurate taxonomic classification.

#### 2.4.2 Microbial Community Composition

Taxonomic classification of the merged reads was performed using Kraken v2.1.2 with the MiniKraken2 database (March 2023 release) on the BV‐BRC microbiome analysis platform and Galaxy.org (usegalaxy.org). Reads were processed in “Single Read Library” mode, and taxonomic assignments were based on exact k‐mer matches to the reference database (Lu et al. 2022).

### 2.5 Data Analysis and Visualisation

Annotated datasets were imported into Python v3.11 and analysed using the pandas, numpy, seaborn, and matplotlib libraries. Exploratory co‐abundance patterns among resistance‐associated annotations and antibiotic target loci were assessed using Spearman correlation coefficients, retaining correlations with ∣r∣≥0.4 and p≤0.05 and visualised in Gephi v0.9.2. Heatmaps and circos plots depicting ARG distributions across samples and taxa were generated using Seaborn and CircosPlot. Resistome and virulome compositions were further summarised using Google Looker Studio and Microsoft Excel. All scripts and pipeline parameters are available upon request.

## 3 Results

Table 1 summarises four publicly available Illumina HiSeq 2000 whole‐genome shotgun metagenomic datasets (M2, M3, M12, and M17) from anaerobic digesters in the USA and Hong Kong, with dataset sizes of 1.4–2.2 Gb and total sequencing output of 3.4–4.7 Gb.

### 3.1 Genome Assembly Characteristics and Annotation Statistics

The four anaerobic digester metagenomic assemblies generated a combined genome length of 138.36 Mb, comprising 12,000 contigs and 146,445 predicted coding sequences (CDSs) (Table 2). Assembly sizes varied across samples, ranging from 22.64 Mb in M3 to 45.66 Mb in M2. The highest number of CDSs was observed in M2 (48,058), whereas the fewest were observed in M3 (23,851).

**TABLE 2:** Genome assembly metrics and chromosomal features of annotated anaerobic digester samples.

Sample ID	GL (bp)	Contig(s)	Contig N50	Contig L50	GC Content	CDS	FP	HP	RNA Genes	CRISPR Genes	pGfam domain	GA	AMR Genes
M2	45,662,946	2805	57,614	201	47.88	48,058	24,169	23,889	675	1634	28,772	7514	348
M3	22,641,346	957	83,331	75	50.99	23,851	12,576	11,275	436	552	13,403	4059	205
M12	37,814,317	6471	20,284	399	53.88	41,047	17,451	23,596	538	775	20,266	6002	276
M17	32,245,798	1767	74,206	108	45.30	33,489	17,924	15,565	556	733	20,609	5532	259
TOTAL	138,364,407	12,000	235,435	783	198	146,445	72,120	74,325	2205	3694	83,050	23,107	1082

Abbreviations: AMR = Antimicrobial resistance; bp = Base pairs; CDS = Coding sequences; Contig(s) = Contiguous sequences of DNA assembled from overlapping reads; FP = Functional proteins; GA = Genome assembly features; GC = Guanine‐cytosine content; GL = Genome length; HP = Hypothetical proteins; L50 = Number of contigs representing 50% of the total genome assembly; M = Sample ID; N50 = Length of the shortest contig at 50% of the total genome assembly; pGfam = Protein family domains.

Assembly quality metrics also differed among samples. Contig N50 values ranged from 20,284 bp (M12) to 83,331 bp (M3), while the number of contigs varied between 957 (M3) and 6471 (M12). GC content ranged from 45.30% in M17 to 53.88% in M12. A total of 72,120 functional proteins and 74,325 hypothetical proteins were predicted across all assemblies.

The four metagenomes contained 2205 RNA genes and 3694 CRISPR‐associated genes. Annotation against antimicrobial resistance databases identified 1082 resistance‐associated genes across all samples, with the highest number detected in M2 (348), followed by M12 (276), M17 (259), and M3 (205) (Table 2).

### 3.2 Functional Gene Distribution Across Anaerobic Digester Metagenomes

Functional annotation revealed that metabolism‐related genes constituted the most abundant subsystem category across all metagenomes, with counts ranging from 3565 in M3 to 6618 in M2 (Table 3). Protein processing was the second most represented functional category, accounting for 1562–2991 genes across samples. Genes associated with energy metabolism were also highly abundant, particularly in M2 (1847) and M12 (1822).

**TABLE 3:** Functional gene distribution across anaerobic digester metagenomes.

Subsystem function	Sample ID			
	M2	M3	M12	M17
Metabolism	6618	3565	4839	4953
Protein processing	2991	1562	2334	2126
Energy	1847	1089	1822	1377
Stress response, defence, virulence	1135	563	869	759
Cellular processes	1721	956	798	1421
Membrane transport	844	574	601	671
DNA processing	1110	510	707	767
RNA processing	735	350	576	524
Cell envelope	266	140	238	178
Regulation and cell signalling	114	78	102	94
Miscellaneous	78	40	54	65

Additional functional categories included cellular processes, DNA processing, stress response, defence and virulence, membrane transport, and RNA processing. M2 generally exhibited the highest abundance across most functional categories, whereas M3 had the fewest annotated genes. Cell envelope functions, regulation and cell signalling, and miscellaneous functions represented the least abundant subsystem categories in all metagenomes.

### 3.3 Classification of Resistance‐Associated Annotations Into Acquired ARGs and Antibiotic Target Loci

The resistance‐associated annotations detected in the anaerobic digester metagenomes were separated into two interpretive categories. The first category included putative acquired ARGs, such as annotations linked to enzymatic inactivation, efflux‐mediated resistance, target protection and other recognised resistance mechanisms. The second category included antibiotic target or housekeeping loci, such as *rpoB*, *rpoC*, *gyrA*, *gyrB*, *EF‐Tu*, *EF‐G*, *Ddl*, *KasA* and *S12p*. These loci are biologically important because they are known antibiotic targets, but their detection alone does not demonstrate resistance unless resistance‐associated mutations, allele‐level variants or linkage to mobile genetic elements are confirmed. Therefore, these loci were interpreted as antibiotic target or resistance‐associated loci rather than definitive acquired ARGs (Table 4).

**TABLE 4:** Antibiotic target and housekeeping loci detected among resistance‐associated annotations in anaerobic digester metagenomes.

Gene	Full name	Function	AMR relevance
*EF‐Tu*	Elongation factor Tu	GTP‐binding protein that brings aminoacyl‐tRNAs to the ribosome during translation.	Involved in protein synthesis; targets translation inhibitors such as kirromycin and pulvomycin; mutations can confer resistance.
*KasA*	β‐Ketoacyl‐ACP synthase A	Involved in the fatty acid synthesis (FAS‐II) pathway, essential for cell wall biosynthesis in mycobacteria.	Targeted by isoniazid and thiolactomycin; resistance via mutation.
*rpoC*	RNA polymerase subunit β'	Catalyses transcription as part of the RNA polymerase complex.	For cell wall synthesis and d‐cycloserine resistance, rpoB and rpoC are involved in rifampicin resistance.
*rpoB*	RNA polymerase subunit β	Catalytic centre for RNA synthesis.	The primary target of rifampicin is mutations that confer rifampicin resistance.
*DDL*	d‐alanine‐d‐alanine ligase	Enzyme in peptidoglycan biosynthesis; forms d‐Ala‐d‐Ala dipeptides for the cell wall.	The target of d‐cycloserine, resistance arises via overexpression or mutation.
*EF‐G*	Elongation factor G	Catalyses the translocation step in protein elongation.	Targeted by fusidic acid; mutations confer resistance.
*MirA*	Multiple antibiotic resistance regulator A (putative)	Thought to regulate efflux systems or envelope integrity.	Putative role in efflux‐mediated AMR; not fully characterised.
*gyrB*	DNA gyrase subunit B	Involved in supercoiling DNA during replication.	Targeted by quinolones and aminocoumarins; mutations confer resistance.
*Iso‐tRNA*	Isoacceptor transfer RNA	Specialised tRNAs that recognise the same amino acid codons.	Not directly conferring resistance, but mutations/modifications can alter translation efficiency. Aminoacylation of tRNA, mupirocin resistance
*S12p*	Ribosomal protein S12	Component of the 30S ribosomal subunit; stabilises the decoding site.	Mutations associated with streptomycin resistance.
*gyrA*	DNA gyrase subunit A	Works with gyrB in DNA supercoiling.	Primary target of quinolones (e.g., ciprofloxacin); mutations confer resistance.

### 3.4 Distribution of Resistance‐Associated Genes and Antibiotic Target Loci

The relative abundance of resistance‐associated loci varied among the four anaerobic digester metagenomes (Figure 1). *EF‐Tu*, *EF‐G*, *rpoB*, *rpoC*, *gyrA*, *gyrB*, *Ddl*, *KasA*, *S12p*, *Iso‐tRNA*, and *MirA* were detected in all samples, although their proportional representation differed. M2 generally exhibited the highest relative abundance for several loci, including *EF‐Tu*, *EF‐G*, and *Iso‐tRNA*, whereas M12 showed increased representation of *rpoB* and *rpoC*. Samples M3 and M17 contributed varying proportions across the detected loci, reflecting differences in resistome composition among the anaerobic digester microbiomes.

**FIGURE 1:**
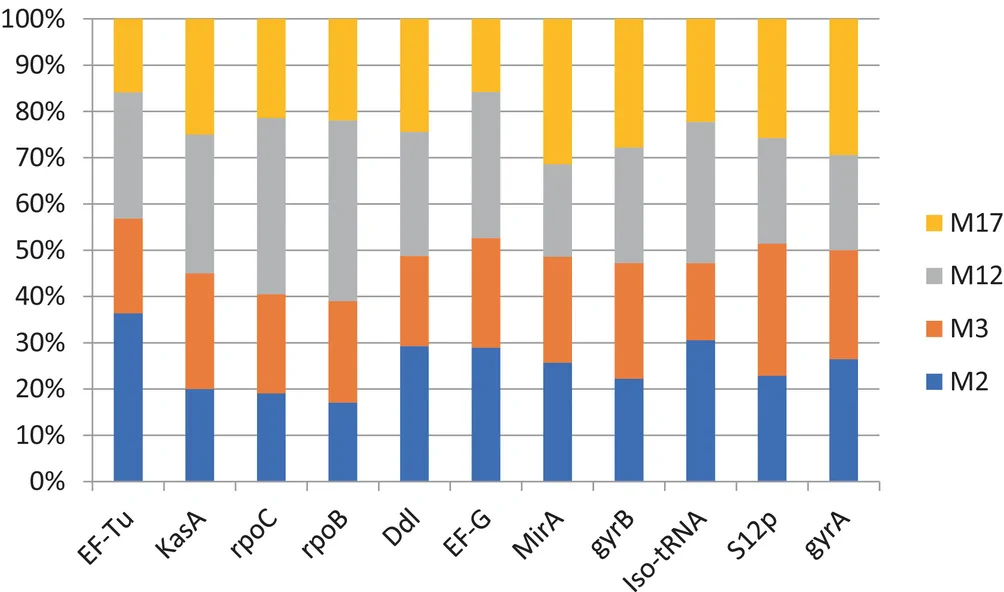
Relative abundance of resistance‐associated loci and antibiotic target genes across anaerobic digester metagenomes.

### 3.5 Distribution of Resistance‐Associated Gene Annotations Across Antibiotic Classes

Resistance‐associated gene annotations were linked to multiple antibiotic classes across all anaerobic digester metagenomes (Figure 2). Aminoglycosides, beta‐lactams, tetracyclines, sulfonamides, quinolones, glycopeptides, peptide antibiotics, and macrolides were among the most frequently represented classes. The relative abundance of antibiotic class‐associated annotations varied among samples, with M2 and M12 generally exhibiting broader representation across multiple antibiotic categories. In contrast, M3 showed comparatively lower representation for several classes (Figure 2).

**FIGURE 2:**
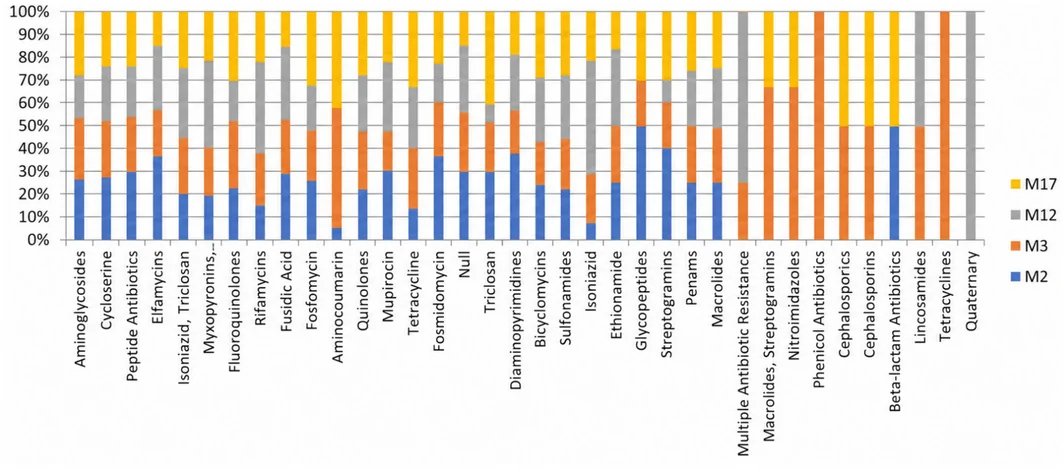
Relative distribution of antibiotic classes associated with resistance‐associated gene annotations across anaerobic digester metagenomes.

### 3.6 Exploratory co‐Abundance Patterns Among Resistance‐Associated and Antibiotic Target Loci

Exploratory correlation analysis was used to examine co‐abundance patterns among the detected resistance‐associated annotations and antibiotic target loci (Figure 3). Strong positive correlations were observed between *rpoB* and *rpoC*, as well as between *gyrA* and *gyrB*. These relationships are biologically expected because rpoB and rpoC encode bacterial RNA polymerase subunits, while *gyrA* and *gyrB* encode DNA gyrase subunits. Therefore, these correlations should not be interpreted as direct evidence of antimicrobial resistance dissemination, co‐selection or horizontal gene transfer. Rather, they likely reflect shared genomic occurrence, functional linkage or similarities in bacterial community composition across the analysed metagenomes.

**FIGURE 3:**
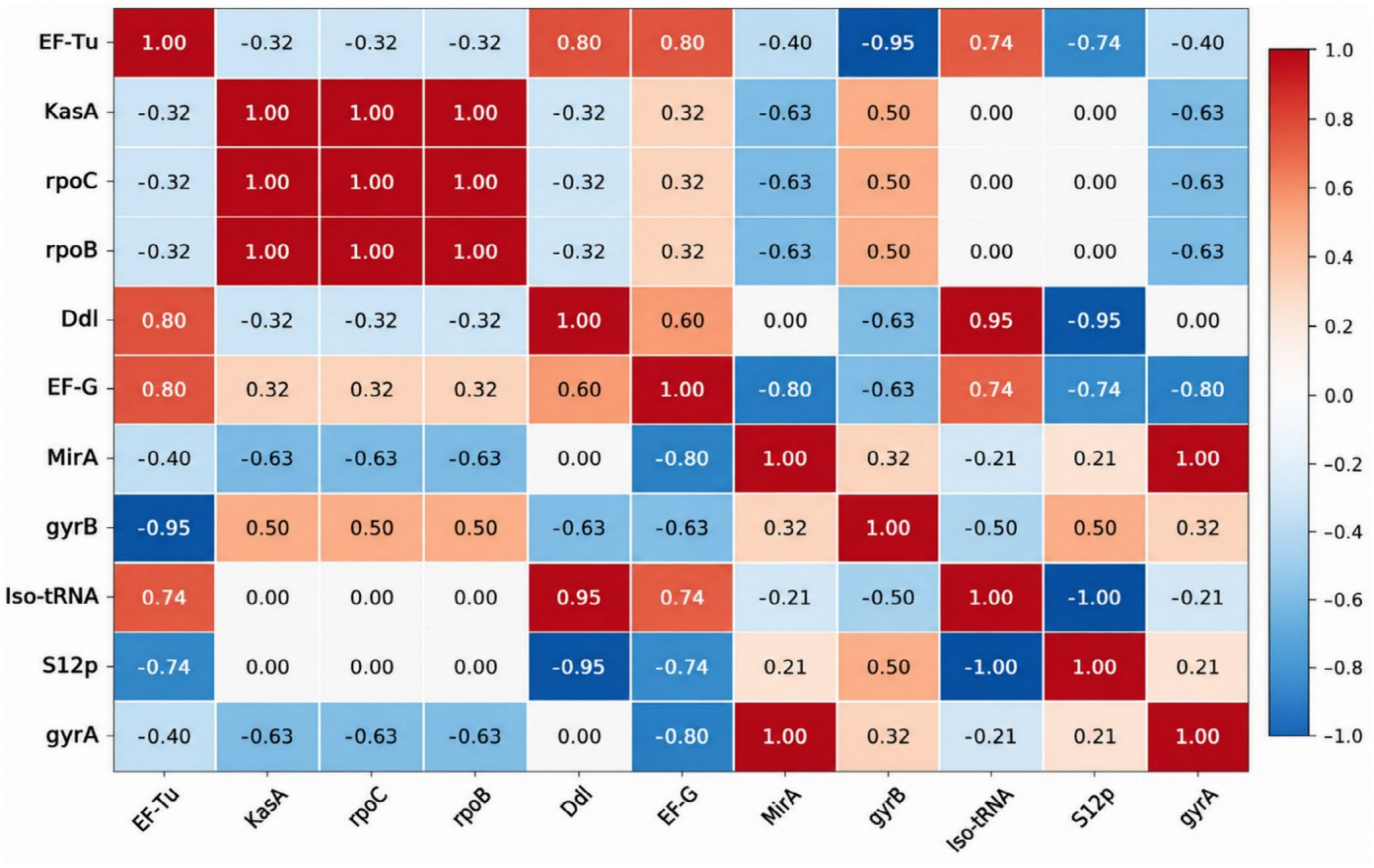
Correlation‐based heatmap showing co‐occurrence patterns among resistance‐associated loci detected in anaerobic digester metagenomes.

## 4 Discussion

### 4.1 Metagenome Assembly Quality and Functional Potential

Anaerobic digestion systems are increasingly recognised not only for their role in renewable energy generation and waste valorisation but also as important microbial ecosystems that may influence the environmental dynamics of antimicrobial resistance (Manyi‐Loh et al. 2019). Using publicly available shotgun metagenomic datasets, this study characterised the genomic features, functional potential, and resistance‐associated gene profiles of anaerobic digester microbiomes. This study's findings revealed substantial variation in assembly quality, functional gene distribution, and resistome composition among samples, reflecting differences in microbial community structure and environmental conditions within anaerobic digestion systems.

The metagenomic datasets analysed differed considerably in sequencing depth, assembly quality, and functional annotation profiles (Tables 1 and 2). Genome sizes ranged from 1.4 to 2.2 Gb, while total sequencing output varied from 3.4 Gb (M12) to 4.7 Gb (M2 and M17). Differences in sequencing strategy appeared to influence assembly outcomes, with paired‐end datasets producing more fragmented assemblies than the more recent Illumina HiSeq datasets. Similar observations have been reported in metagenomic investigations where sequencing depth, library preparation, and read quality significantly influenced assembly continuity and downstream gene recovery (Sanders et al. 2019).

Assembly statistics revealed substantial heterogeneity among samples. Genome lengths ranged from 22.6 to 45.6 Mb, while contig numbers varied from 957 (M3) to 6471 (M12). The highest assembly contiguity was observed in M3 (N50 = 83.3 kb), whereas M12 exhibited the lowest contiguity (N50 = 20.3 kb), confirming the influence of sequencing characteristics on assembly performance. The GC content ranged from 45.3% to 53.9%, suggesting considerable taxonomic diversity within the anaerobic digester microbiomes. According to the study by Player et al. (2021), this variation is consistent with previous investigations of biogas‐producing systems, in which microbial community composition strongly influences genomic characteristics and functional potential.

Moreover, coding sequences were abundant across all metagenomes, with M2 containing the highest number. Furthermore, a substantial proportion of proteins were classified as hypothetical proteins, indicating that a significant fraction of the microbial functional potential within anaerobic digesters remains uncharacterised. This observation aligns with previous environmental metagenomic studies that reported large pools of poorly annotated genes with potential ecological and biotechnological significance (Rahman et al. 2022; Chevallereau et al. 2022).

As shown in Table 3, similar functional profiles have been reported in biogas‐producing systems, where metabolic functions consistently account for a large proportion of annotated genes and support the sequential hydrolytic, fermentative, acetogenic, and methanogenic stages of anaerobic digestion (Pyzik et al. 2018). Moreover, in this study, protein processing and energy‐associated genes were also highly represented, particularly in M2. These functions are essential for maintaining microbial growth, protein turnover, and metabolic efficiency under the dynamic environmental conditions characteristic of anaerobic digesters. The enrichment of energy metabolism genes is consistent with the energy‐intensive biochemical pathways required for methane generation and organic matter transformation (Badra et al. 2025).

Genes associated with stress response, defence, and virulence were abundant across all samples. Their occurrence likely reflects microbial adaptation to environmental stressors, including fluctuating substrate composition, accumulation of inhibitory metabolites, nutrient competition, and microbial antagonism. Previous studies have similarly reported enrichment of stress‐related genes in waste‐treatment ecosystems exposed to diverse environmental pressures.

In Table 2, resistance‐associated gene annotations were detected across all anaerobic digester metagenomes, with M2 exhibiting the highest abundance, followed by M12, M17, and M3. The diversity of resistance‐associated annotations observed in this study supports previous reports that anaerobic digestion systems can serve as reservoirs of genetic elements linked to antimicrobial resistance pathways (Nnadozie et al. 2017).

Annotations were associated with multiple antibiotic classes, including aminoglycosides, beta‐lactams, tetracyclines, quinolones, macrolides, glycopeptides, and sulfonamides (Figure 2). The widespread occurrence of annotations linked to these antibiotic classes may reflect the diverse microbial populations inhabiting anaerobic digesters and the selective pressures exerted by waste‐derived substrates. Similar patterns have been reported in wastewater treatment plants, agricultural waste systems, and other environmental reservoirs of antimicrobial resistance (Levy and Marshall 2004).

Functional classification identified several resistance‐associated loci and antibiotic target genes, including EF‐Tu, KasA, rpoC, rpoB, Ddl, EF‐G, MirA, gyrB, Iso‐tRNA, S12p, and gyrA (Table 4 and Figure 1). These genes are involved in essential cellular processes, including translation, transcription, DNA replication, and cell wall biosynthesis (Pan et al. 2022). Importantly, this study identified these loci through homology‐based functional annotation and did not assess resistance‐conferring mutations at the nucleotide level. Consequently, their detection should be interpreted as evidence of resistome potential and the presence of antibiotic target loci rather than confirmation of phenotypic antimicrobial resistance.

A key interpretive issue in environmental metagenomic resistome studies is the distinction between acquired ARGs and antibiotic target or housekeeping genes. Acquired ARGs may directly encode resistance functions such as drug inactivation, efflux, target protection or target replacement. In contrast, target loci such as rpoB, gyrA, gyrB and EF‐Tu are present in many bacterial genomes as part of normal cellular function, and only confer resistance when specific mutations or resistant alleles are present. Since the present study did not perform mutation‐level variant calling, the detection of these loci was interpreted cautiously as resistance‐associated or antibiotic target potential rather than direct evidence of phenotypic AMR.

Several of the detected loci have been previously associated with antimicrobial resistance through specific mutations. For example, mutations in rpoB have been linked to rifampicin resistance, while alterations in gyrA and gyrB contribute to fluoroquinolone resistance (Álvarez‐Aldana et al. 2026). Likewise, mutations affecting EF‐Tu, Ddl, and KasA have been implicated in resistance to specific antimicrobial classes. However, because mutation‐level analyses were beyond the scope of the present investigation, the functional significance of these loci within the studied metagenomes remains to be established.

The widespread occurrence of resistance‐associated gene annotations across the anaerobic digester metagenomes confirms the potential role of anaerobic digestion systems as environmental reservoirs of antimicrobial resistance‐related genetic elements. Annotations linked to multiple antibiotic classes, including aminoglycosides, beta‐lactams, tetracyclines, quinolones, macrolides, glycopeptides, and sulfonamides, were detected across all samples (Figure 2). The persistence of these annotations within anaerobic digestion systems is of particular interest because digestate products are frequently applied to agricultural soils, creating potential pathways for the environmental dissemination of resistance‐associated genetic material (Allegrini and Zabaloy 2024).

Several of the detected loci, including rpoB, rpoC, gyrA, gyrB, EF‐Tu, EF‐G, Ddl, KasA, S12p, and MirA, have previously been associated with resistance mechanisms or antibiotic target pathways in clinically and environmentally relevant microorganisms (Table 4) (Benamri et al. 2021). These loci participate in essential cellular functions, including transcription, translation, DNA replication, and cell wall biosynthesis. Their detection across multiple metagenomes suggests that anaerobic digester microbial communities harbour a diverse repertoire of genes involved in biological processes that may influence antimicrobial susceptibility and adaptation under selective environmental pressures.

The identified annotations were linked to antibiotic classes widely used in human medicine, veterinary practice, and agriculture. Similar resistance‐associated profiles have been reported in wastewater treatment plants, livestock waste management systems, and agricultural environments, where antimicrobial residues and resistant microorganisms contribute to the maintenance and spread of resistance‐related genetic elements (Anand et al. 2021). The presence of annotations associated with beta‐lactams, tetracyclines, aminoglycosides, and sulfonamides is particularly noteworthy, as these antimicrobial classes remain among the most widely used worldwide and are frequently included in resistance surveillance programmes.

Based on CARD annotation and supporting literature, several resistance‐associated loci detected in this study have previously been reported in bacterial taxa, including *Escherichia coli, Klebsiella pneumoniae*, *Pseudomonas aeruginosa* , *Enterococcus faecium* , *Clostridium species*, *Bacillus subtilis* , and *Mycobacterium tuberculosis* . These host associations provide valuable biological context for interpreting the detected annotations. However, because the present study did not perform taxonomic binning of individual loci, host‐resolved metagenomic reconstruction, or contig‐level linkage analysis, these associations should not be interpreted as direct evidence that the detected genes originated from these specific taxa within the analysed anaerobic digesters.

Further, correlation‐based co‐occurrence analysis revealed distinct patterns of association among the resistance‐associated loci detected across the anaerobic digester metagenomes (Figure 3). Several loci exhibited strong positive correlations, including rpoB‐rpoC and gyrA‐gyrB, indicating similar abundance patterns across the analysed samples. These associations are not unexpected, as the genes encode components of the same essential cellular machinery. Specifically, rpoB and rpoC encode subunits of bacterial RNA polymerase, whereas gyrA and gyrB encode subunits of DNA gyrase, both of which function cooperatively during transcription and DNA replication, respectively (Peng et al. 2024).

The strong positive associations observed among these loci are therefore more likely to reflect shared genomic occurrence, common taxonomic distribution, or functional interdependence rather than direct evidence of antimicrobial resistance dissemination. Similar co‐abundance patterns have been reported in environmental metagenomes, where genes involved in related biological processes frequently exhibit correlated abundance due to their co‐presence within microbial genomes or ecological guilds (Goberna et al. 2019; Cheng et al. 2024). Consequently, the correlations identified in this study should be interpreted as exploratory indicators of gene co‐occurrence rather than proof of co‐selection, horizontal gene transfer, or mobile genetic element‐mediated spread.

Additional positive correlations were observed between Ddl and Iso‐tRNA, suggesting coordinated occurrence of genes involved in translation and cell wall biosynthesis. Such relationships may reflect common physiological requirements for microbial growth and adaptation within anaerobic digestion systems. Previous studies have demonstrated that genes participating in complementary metabolic or cellular pathways often display correlated abundance patterns within complex microbial communities (Peng et al. 2024).

Conversely, several loci displayed negative correlations, including EF‐Tu and gyrB, as well as EF‐G and MirA (Benítez‐Páez et al. 2014). These inverse relationships may indicate differences in taxonomic distribution, ecological niche preference, or selective pressures acting on distinct microbial populations. Similar negative associations have been reported in microbial communities exposed to varying environmental conditions, where different functional strategies contribute to adaptation and survival (Lu et al. 2016). However, because the present analysis was based solely on abundance correlations, the biological significance of these negative associations remains uncertain and warrants further investigation.

The co‐occurrence heatmap (Figure 3), therefore, provides a useful overview of the abundance relationships among resistance‐associated loci within anaerobic digester microbiomes. Nevertheless, the analysis does not establish physical linkage of genes on the same contigs, plasmids, integrons, or other mobile genetic elements. Neither does it demonstrate the presence of resistance‐conferring mutations within the detected loci.

## 5 Conclusion and Recommendation

This study re‐analysed four publicly available anaerobic digester shotgun metagenomes and showed that these systems harbour diverse functional genes, resistance‐associated annotations and antibiotic target loci. The findings support the view that anaerobic digesters may contribute to the environmental resistome, especially where digestate is reused in agricultural settings. However, the results should be interpreted cautiously because several frequently detected loci, including rpoB, rpoC, gyrA, gyrB, EF‐Tu and EF‐G, are housekeeping or antibiotic target genes rather than definitive acquired ARGs. Their presence alone does not confirm antimicrobial resistance without mutation‐level, allele‐level or mobile genetic element evidence.

Future studies should integrate acquired ARG‐focused profiling, host‐resolved metagenomic reconstruction, plasmid and integron detection, contig‐level ARG‐MGE linkage and variant‐level analysis of antibiotic target genes. Such approaches will be necessary to determine whether resistance‐associated annotations in anaerobic digesters represent transferable environmental AMR risks or reflect the background genomic composition of resident microbial communities. Routine monitoring of anaerobic digestion systems and digestate products is recommended within a One Health surveillance framework, particularly where digestate is applied to agricultural soils.

## Author Contributions

**Oluwaseyi Paul Olaniyan:** conceptualization, investigation, methodology, writing – review and editing. **Elijah Kolawole Oladipo:** supervision, analysis, writing – review and editing. **Omokaro Obire:** conceptualization, supervision, writing – review and editing. **Fatimah Adenike Ajadi:** methodology, data curation. **Emmanuel Sunday Fajoyegbe:** methodology, investigation, analysis. **Ademola Ayodele Adesoye:** methodology, resources. **Olamilekan Emmanuel Oyinloye:** methodology, data curation. **Olasunbo Aina Olasupo:** analysis, writing – original draft, investigation. **Sunday Babatunde Akinde:** conceptualization, writing – review and editing, project administration, supervision, resources, methodology, visualization. **Olabisi Ojo:** conceptualization, supervision. **Adedolapo Aishat Salami:** methodology, data curation. **Adejoke Janet Ajani:** methodology. **Waidi Folorunso Sule:** writing – review and editing, supervision.

## Funding

The Authors received no external funding for this study.

## Ethics Statement

The authors have nothing to report.

## Conflicts of Interest

The authors declare no conflicts of interest.

## Data Availability

The data that support the findings of this study are openly available in National Center for Biotechnology Information (NCBI) Genbank at https://www.ncbi.nlm.nih.gov/.
